# Exposure to endosulfan can cause long term effects on general biology, including the reproductive system of mice

**DOI:** 10.3389/fgene.2022.1047746

**Published:** 2022-11-24

**Authors:** Anju Sharma, Arigesavan Kaninathan, Sumedha Dahal, Susmita Kumari, Bibha Choudhary, Sathees C. Raghavan

**Affiliations:** ^1^ Department of Biochemistry, Indian Institute of Science, Bangalore, India; ^2^ Institute of Bioinformatics and Applied Biotechnology, Bangalore, India

**Keywords:** endosulfan, genomic stability, infertility, spermatogenesis, oogenesis, reproductive toxicity, double-strand break, DNA repair

## Abstract

Increased infertility in humans is attributed to the increased use of environmental chemicals in the last several decades. Various studies have identified pesticides as one of the causes of reproductive toxicity. In a previous study, infertility was observed in male mice due to testicular atrophy and decreased sperm count when a sublethal dose of endosulfan (3 mg/kg) with a serum concentration of 23 μg/L was used. However, the serum concentration of endosulfan was much higher (up to 500 μg/L) in people living in endosulfan-exposed areas compared to the one used in the investigation. To mimic the situation in an experimental setup, mice were exposed to 5 mg/kg body weight of endosulfan, and reproductive toxicity and long-term impact on the general biology of animals were examined. HPLC analysis revealed a serum concentration of ∼50 μg/L of endosulfan after 24 h endosulfan exposure affected the normal physiology of mice. Histopathological studies suggest a persistent, severe effect on reproductive organs where vacuole degeneration of basal germinal epithelial cells and degradation of the interstitial matrix were observed in testes. Ovaries showed a reduction in the number of mature Graafian follicles. At the same time, mild vacuolation in liver hepatocytes and changes in the architecture of the lungs were observed. Endosulfan exposure induced DNA damage and mutations in germ cells at the molecular level. Interestingly, even after 8 months of endosulfan exposure, we observed increased DNA breaks in reproductive tissues. An increased DNA Ligase III expression was also observed, consistent with reported elevated levels of MMEJ-mediated repair. Further, we observed the generation of tumors in a few of the treated mice with time. Thus, the study not only explores the changes in the general biology of the mice upon exposure to endosulfan but also describes the molecular mechanism of its long-term effects.

## Introduction

Pesticides are applied in the environment to increase agricultural yield by controlling pests. Currently, ∼2 million tonnes of pesticides are utilized around the globe each year, which has been estimated to increase to 3.5 million tonnes by 2020 (Eddleston et al., 2002; Zhang, 2018; Sharma et al., 2019). Organochlorine pesticides like DDT (Dichlorodiphenyltrichloroethane) and BHC (Benzene hexachloride) are one of the earliest chemical pesticides used around the world (Simonich and Hites, 1995; Pimentel, 1996; Vijgen et al., 2011). Due to their persistence and long-range transport, they were replaced with the less persistent organophosphate and carbamate pesticides (Goff and Giraudo, 2019). Nevertheless, the residue of organochlorine pesticides is still detected in the environment in many countries where it is not at all used. Moreover, developing countries, including several Asian countries, still use these pesticides (Iwata et al., 1993; Senior, 2009; Gill et al., 2020). Thus, investigating the health and environmental effect of these pesticides are still relevant and necessary.

Endosulfan (6, 7, 8, 9, 10, 10-hexachloro-1, 5, 5a, 6, 9, 9a-hexahydro-6, 9-methanol-2, 4,3-benzadioxathiepin 3-oxide) is one of the major cyclodiene pesticides, which is in continuous use since the 1950s. Although banned in several western countries by the late 2010s (Vandenberg et al., 2020), endosulfan (ES) is still used in developing countries. The WHO classified it as Class II (moderately hazardous), while US EPA and EU have classified it as Class I (highly acutely toxic) (ATSDR, 2000a; Atsdr, 2000b; World Health, O., and International Programme on Chemical, S, 2010).

Commercially, ES is a mixture of two isomeric forms, *α*- and *β*-Endosulfan, in a ratio of 3:1 (Carrera et al., 2002; Singh et al., 2014). In mammalian systems, it is metabolized into the most persistent and toxic metabolite, endosulfan sulfate, and endosulfan diol, which is further metabolized into endosulfan ether, hydroxy ether, and lactone (Kataoka and Takagi, 2013). Endosulfan and its related isomers were listed as persistent organic pollutants due to their high toxicity, bioaccumulation, long residual period in soil, and long-distance transportation under the Stockholm Convention held in May 2011 (UNEP, 2011). It is persistent in the environment, taking decades to biodegrade. Endosulfan is frequently detected in river water in Africa, Europe, and Asian countries, including India (Dalvie et al., 2003; Hellar-Kihampa et al., 2013; Loha et al., 2020; Montuori et al., 2020). A study showed that a concentration of *α*- and *β*- ES ranged from non-detectable limit to 35.21 μg/L and 37.56 μg/L, respectively, in Tapi river water in India (Sarker et al., 2021). The mean concentration of ES in the river Ganga in India was ∼36 ng/L (Khuman and Chakraborty, 2019). Some of its initial degradation products are structurally similar to parent endosulfan isomers and share the same toxic effects (Dorough et al., 1978).

A previous study from our laboratory has shown that exposure to ES in mice (3 mg/kg body weight) induces an increase in DNA damage, triggers compromised DNA damage response, and promoted the error-prone microhomology-mediated end-joining (MMEJ) pathway of DNA repair in the male reproductive organ (Sebastian and Raghavan, 2016). ES-induced DNA damage was highest in the lungs and testes when analyzed through immunohistochemistry, immunofluorescence, and Western blot analysis (Sebastian and Raghavan, 2015a; Sebastian and Raghavan, 2015b; Sebastian and Raghavan, 2016; Sebastian and Raghavan, 2017). TUNEL assay revealed DNA damage in spermatogonia mother cells, Sertoli cells, and primary spermatocytes in the same study. The potential of ES to induce DNA damage and compromised DNA repair mechanisms in germ cells is critical for fertility and the stable propagation of species. In the same study, when a sublethal dose of ES was used with a serum concentration of 23 μg/L after 24 h, male mice (33%) infertility was observed due to testicular atrophy. It decreased sperm count and increased mortality (Sebastian and Raghavan, 2015b). However, the serum concentration of ES is much higher in people living in highly exposed areas, with a concentration range of 8.85–547.6 μg/L ES in the affected human population, which can be 700 μg/L after 2 h of exposure (Mnif et al., 2011; Sebastian and Raghavan, 2015b).

ES poisoning increases the incidence of neurological complications and congenital and reproductive abnormalities among the affected people (James and Emmanuel, 2021). However, the effect of ES on the female reproductive system was not investigated previously. Epidemiological studies show that farmers exposed to ES were more prone to developing tumors (Zahm and Ward, 1998) and multiple myeloma (Khuder and Mutgi, 1997). Similarly, people exposed to ES were reported to have an increased risk of developing various cancers in their life (Melangadi, 2017). Although the immediate effects of pesticides are studied extensively, their delayed actions are not explored at the molecular level. There is a knowledge gap between pesticide exposure and its long-term effects on humans. Therefore, to investigate the long-term impact of ES in an exposed human population, we have used a mice model system, considering the relevant concentrations of ES in the exposed areas. In the present study, we report that exposure to ES leads to significantly compromised fertility in both male and female mice. Further, we find a long-term persistent effect on the histopathology of reproductive tissues, liver and lung. Persistent elevated levels of DNA damage leading to compromised DNA repair were also observed. Further, we report an increase in the incidence of tumor development in the mice exposed to ES in their later life.

## Materials and methods

### Chemicals, reagents, and antibodies

Endosulfan (pestanal) was purchased from Millipore-Sigma (Catalogue No. 32015). Other chemicals and reagents were obtained from Millipore-Sigma (St. Louis, MO, United States) or Sisco Research Laboratories Ltd. (SRL), India. Antibodies were procured from Santa Cruz Biotechnologies (United States) and BD Biosciences (United States).

### Animals

All animal experiments were carried out with the approval of the animal ethical committee of the Indian Institute of Science (IISc), Bangalore, India (CAF/Ethics/228/2013; CAF/Ethics/793/2020). Balb/c mice of 4–6 weeks old weighing 20 g–25 g were purchased from Central Animal Facility (CAF), IISc. The animals were housed in polypropylene cages and provided a standard pellet diet (21% protein, 5% lipids, 4% crude fiber, 8% ash, 1% calcium, 0.6% phosphorous, 3.4% glucose, 2% vitamin, and 55% nitrogen-free extract (carbohydrates) and purified water *ab libitum*. The mice were maintained under controlled temperature and humidity conditions with a 12 h light/dark cycle.

### Administration of endosulfan in mice and hematological analysis

ES was dissolved in 0.05% methylcellulose, and mice were orally fed using a gastric gavage (Sebastian and Raghavan, 2015b). The treatment consisted of a dose of 5 mg/kg on every alternative day for 10 doses.

After 31 days of ES exposure (5 mg/kg, 10 doses), two animals from the treated and control groups were sacrificed using CO_2_ asphyxiation. Blood was collected in EDTA-coated vials by heart puncture. Analysis of blood samples was performed at Rohana Veterinary Diagnostic Lab, Bangalore, India. Red blood cells (RBC), white blood cells (WBC), platelet counts (PLT), packed cell volume (PCV), hemoglobin (HGB), mean cell volume (MCV), mean corpuscular hemoglobin (MCH), mean corpuscular hemoglobin concentration (MCHC), lymphocytes, neutrophils, eosinophils, and monocytes were analyzed as described before (Sharma et al., 2020; Sharma et al., 2021).

### Serum biochemistry

After 31 days of ES exposure (5 mg/kg, 10 doses), two animals from the treated and control groups were sacrificed using CO_2_ asphyxiation. Blood was collected in tubes without anticoagulant and centrifuged (900 ×*g*, 10 min) for serum extraction. Serum samples were analyzed at Rohana Veterinary Diagnostic Lab, Bangalore, India. Liver and kidney function tests such as serum glutamic pyruvic transaminase (SGPT), serum glutamic oxaloacetic transaminase (SGOT), alkaline phosphatase (ALP), albumin, total protein, bilirubin, blood urea nitrogen (BUN), creatinine, uric acid, and phosphorous were analyzed as described earlier (Sebastian and Raghavan, 2015b; Vartak et al., 2016; Sharma S. et al., 2020).

### Impact of endosulfan exposure on fertility

Mating experiments in Balb/c mice were performed as described before (Chiruvella et al., 2012; Sebastian and Raghavan, 2015b). After 7 days post-completion of ES treatment, 1:2 (male: female) mating was set up in separate cages for 10 days or two oestrus cycles. At the end of the 10th day, males and females were separated, and the females were observed for pregnancy in the following days. A male was considered infertile if all two females in the cage failed to get impregnated. Four groups of animals were maintained for mating; 1) treated male and untreated female; 2) treated female and untreated male; 3) treated male and treated female; 4) control group comprising untreated male and untreated female (*n* = 5/group for male and *n* = 10/group for female). The experiments were repeated three independent times on separate occasions.

### Histopathological evaluation

Mice organs (brain, lungs, liver, ovary, testes, intestine, and spleen) collected from the animals after 8 months of ES treatment were processed as per standard protocol (Srivastava et al., 2012; Sharma et al., 2013; Gopalakrishnan et al., 2021) and embedded in paraffin. Microtome sectioning was done using rotary microtome with a section thickness of 5 µm (Leica Biosystems, Germany) and stained with hematoxylin and eosin. Images were captured using a bright field microscope (Carl Zeiss, Oberkochen, Germany). Tissues from two mice were subjected to histopathological analysis a minimum of two independent times. The veterinarians analyzed histopathological results at Central Animal Facility, IISc, Bangalore.

### Immunofluorescence analysis

Immunofluorescence studies were performed as described before (Chiruvella et al., 2012; Kumari et al., 2019). Paraformaldehyde fixed paraffin embedded tissues sectioned at 5 µm thickness were de-paraffinized and rehydrated in a series of alcohol gradients. Antigen retrieval was done in 10 mM sodium-citrate buffer (pH 6.0), followed by blocking in PBST containing 1% BSA and 10% FBS. Primary antibody [53BP1 (SC 22760) dilution 1:100 and Ligase III (BD 611876) dilution 1:500]; incubation was carried out overnight at 4^°^C. Slides were washed and incubated with appropriate Alexa Fluor conjugated secondary antibody for 3 h at room temperature. After washing the slide, it was mounted with DAPI-DABCO, and images were taken in a confocal microscope (Olympus, FLUOVIEW FV3000, Japan) and processed by Olympus software. The experiments were repeated independently with multiple technical repeats on separate occasions.

### Pharmacokinetics of endosulfan

ES was administered orally to Balb/c mice (5 mg/kg, one dose). Animals were sacrificed at 4, 8, 10, 12, 24, and 48 h post-administration, deproteinization was done using acetonitrile, and the sera were used for HPLC analysis (Shimadzu, Kyoto, Japan) (Srivastava et al., 2012; Sebastian and Raghavan, 2015b; Sharma et al., 2021). Liquid Chromatography (LC) was carried out using a C18 analytical column (Shimadzu). The mobile phase consisted of an isocratic mixture of acetonitrile and water at 70:30 v/v. Spectra were acquired at 214 nm. The injection volume was 20 μl (automatic), and the flow rate used was 0.5 ml/min. The standard solution of ES was prepared by diluting the stock to 1, 5, and 10 μM of the compound in the serum. Standard calibration curves were plotted using peak area against the concentration and retention time of the compound, were also determined. Plasma concentrations of the treated groups were extrapolated from standard curves. Pharmacokinetic parameters were analyzed using LabSolutions software (Shimadzu, Japan). The values obtained were plotted with GraphPad Prism (ver5.1) software, where “C” is the predicted concentration, and “t” is time. Data were analyzed using nonlinear regression analysis. Maximum drug plasma concentration (C_max_) and time to reach maximum concentration T_max_ were determined by the area under the curve versus the time curve. The clearance rate (CL) was calculated by the rate of drug elimination in plasma (Vartak et al., 2016; Sharma et al., 2021).

### Quantification and statistical analysis

All quantifications were performed using GraphPad Prism software, and statistical significance (mean ± SEM) was calculated. All statistical analyses were performed in GraphPad Prism software using one-tailed unpaired student’s *t*-tests. Error bars have been shown depicting mean ± SEM (ns: not significant, *p < 0.05, **p < 0.005, ***p < 0.0001). Colocalization analysis was done using JaCoP in ImageJ software. The values were plotted in GraphPad Prism 5.0, and the significance was calculated using the same.

## Result

### The bioavailability of endosulfan increases with an increased dose

**FIGURE 1:**
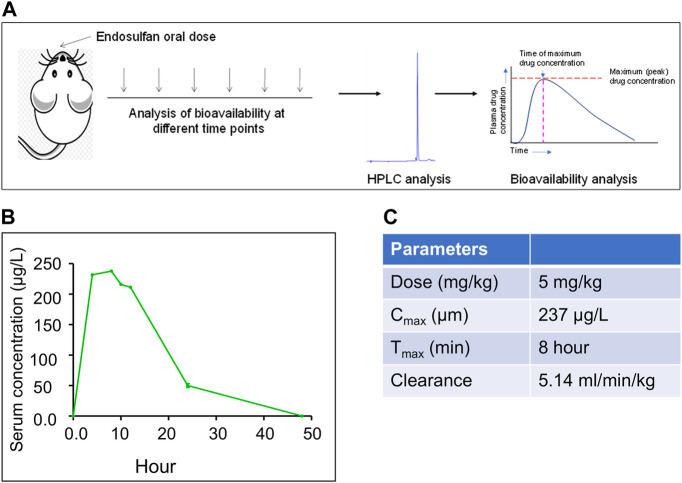
Evaluation of bioavailability of ES in mice when exposed to 5 mg/kg body weight of ES. **(A)** Schematic of the experimental strategy. HPLC analysis was performed using serum samples collected at different time points 4, 8, 10, 12, 24, and 48 h after oral ingestion of ES (5 mg/kg body weight, *n* = 3). **(B)** *In vivo* bioavailability analysis of ES in the serum of Balb/c mice at 4, 8, 10, 12, 24, and 48 h after exposure with ES (5 mg/kg body weight). Three animals were sacrificed for each time point, sera were collected and subjected to HPLC analysis at 214 nm wavelength. **(C)** Pharmacokinetics of ES in mice serum. The area under the curve (AUC) was calculated to obtain the maximum circulating concentration (C_max_) and maximum time (T_max_) to reach C_max_.

The appropriate concentration of ES for analysis of physio-molecular changes in mice model system, which can be correlated with humans, is important for the investigation of its effect on human health as exposure to it is reported to cause abnormalities and congenital disabilities in areas of extensive use (Satheesh, 2017; James and Emmanuel, 2021). Since the people living in ES-exposed areas showed a serum concentration of up to 550 μg/L of the pesticide, our study selected a concentration that can mimic the situation. Mice were treated with 5 mg/kg body weight of endosulfan based on the previous investigation (Sebastian and Raghavan, 2015b) (Figure 1A). HPLC analysis was performed to ascertain the serum concentration of ES in mice by collecting serum samples at different time points (4, 8, 10, 12, 24, and 48 h) after oral ingestion (Figure 1B). For standard, serum samples from control mice were collected and spiked with different concentrations of ES. Our results show that the serum concentration of ES after 24 h was ∼50 μg/L compared to the previously observed 23 μg/L when 3 mg/kg of ES was used (Sebastian and Raghavan, 2015b) (Figure 1B).

HPLC analysis post oral dose of ES revealed that the maximum concentration of ES (C_max_) was 237 μg/L and the time to reach C_max_, i.e., T_max_, was 8 h (Figure 1C). We observed the clearance rate (CL) of 5.14 ml/min/kg in mice serum based on the elimination rate in plasma (Figure 1C). It was eliminated from the blood at the rate of 5.14 ml/min/kg. We did not detect ES in serum after 48 h of oral ingestion.

### Exposure to endosulfan affects the normal physiology of mice

**FIGURE 2:**
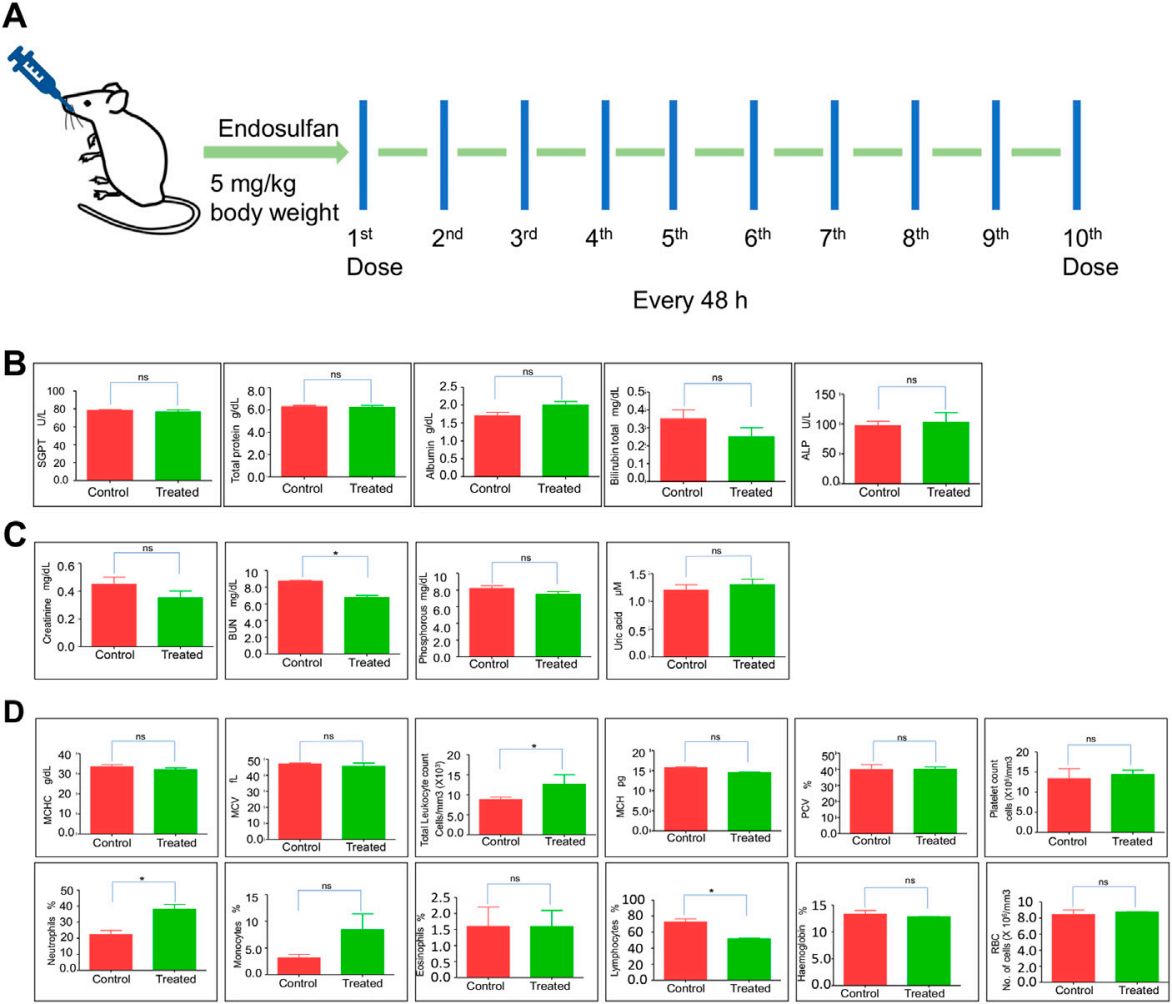
Evaluation of physiological effects of ES in mice. **(A)** Schematic representation of ES-treatment in mice. Mice were exposed to 5 mg/kg body weight of ES through oral gavage every alternate day for 10 doses. **(B,C)** Liver and kidney function test of ES-treated mice. Bar graphs indicating enzymatic activities reflective of liver and kidney function at 31st day after ES treatment (5 mg/kg, *n* = 2). **(D)** Blood parameter analysis following oral administration of ES in mice after 31st day (*n* = 2). Analysis of red blood cells (RBC), white blood cells (WBC), haemoglobin (HGB), platelets, MCHC, MCV, MCH, PCV, neutrophils, monocytes, eosinophils, and lymphocytes counted among control and treated after 31 days of administration. Error bars denote mean ± SEM (ns: not significant, *p < 0.05).

Evaluation of biochemical changes in organs is essential to understand the effects of xenobiotics. Therefore, we were interested in evaluating the impact of exposure when 5 mg/kg (10 doses) of ES was used. To do this, we performed liver and kidney function tests after 31 days of ES treatment (Figure 2A). Results showed no significant difference between the control and treated mice in the level of SGPT, total protein, albumin, bilirubin, and ALP (Figure 2B). However, analysis of kidney function (creatinine, phosphorous, uric acid, and BUN levels) showed a significant decrease in BUN levels indicating liver and kidney injury (Figure 2C). This result suggests that ES affects the function of the kidney and liver.

Hematological parameters are routinely used to indicate the physiological or sublethal stress responses to endogenous and exogenous changes. To analyze any biochemical changes, we assessed different blood parameters in ES-exposed mice (5 mg/kg) after 31 days compared to the control mice (Figure 2D). The quantitative analysis showed a significant increase in total leukocyte counts and neutrophils percentage, possibly due to inflammation but a significant decrease in lymphocyte percentage compared with the controls, indicating that the mice were under stress (Figure 2D). Although an increase in the monocyte population was observed, it was not significant. Blood parameters like platelet counts, RBC, MCHC, MCV, Eosinophils, MCH, and PCV remained unchanged. These results indicate that ES alters the hematological and biochemical parameters in mice.

### Endosulfan induces the reproductive toxicity

**FIGURE 3:**
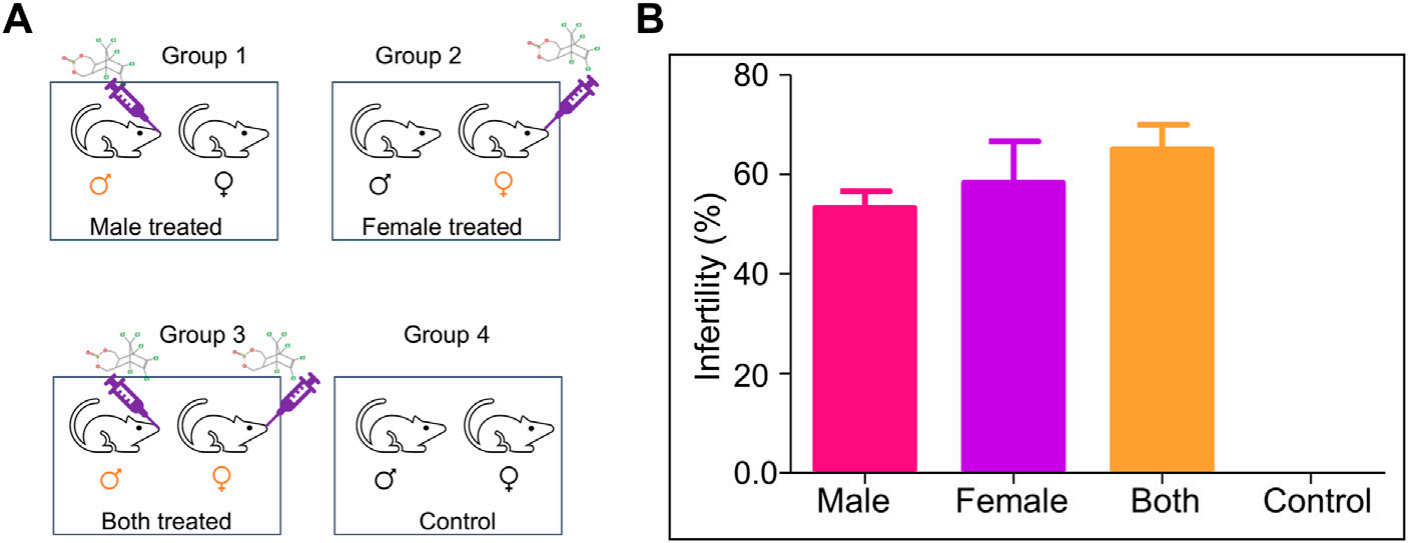
Evaluation of the effect of ES on fertility in mice. **(A)** Schematic is showing mating groups following exposure to endosulfan (5 mg/kg body weight); Group 1 (male treated), Group 2 (female treated), Group 3 (male and female treated), and Group 4 (untreated control). **(B)** Bar graphs show the difference in fertility levels when ES was given only to males (*n* = 5), only to females (*n* = 10), or to both males and females (*n* = 15). Mating was in the ratio of 1:2 of male to female. Experiments were repeated 3 times. Error bars denote mean ± SEM.

Mating experiments (1:2 ratio, male: female) were set up the post-one week of the last dose of ES treatment (5 mg/kg, 10 doses) in the mice. The mating was set up in four different groups, Group 1, where ES-exposed male mice were mated with normal female mice; Group 2, where ES-exposed female mice were mated with normal male mice; Group 3, where both male and female mice were exposed to ES and Group 4 was the untreated control group (Figure 3A).

Male mice were considered infertile if they could not impregnate any female mice. In contrast, female mice were considered infertile if it was not able to get pregnant even after the mating period of 10 days. The percentage of infertility in Group 1 (only male treated) was 55%, while in Group 2 (only female treated), it was 62%. The most severe effect was observed when both parents were treated (67% infertility). However, no infertility was observed when untreated mice were subjected to mating (Figure 3B). Therefore, a significant increase in infertility was observed in the present study compared to the previous report (Sebastian and Raghavan, 2015a).

### Endosulfan affects the reproductive organs of mice even after several months of exposure

Although the acute effect of pesticides is studied in detail, its long-term effects are not followed up systematically. When we evaluated the mice for long-term effects of pesticide exposure, we did not find any difference in food and water intake between control and ES-exposed mice. Besides, their social behaviour remained unchanged.

**FIGURE 4:**
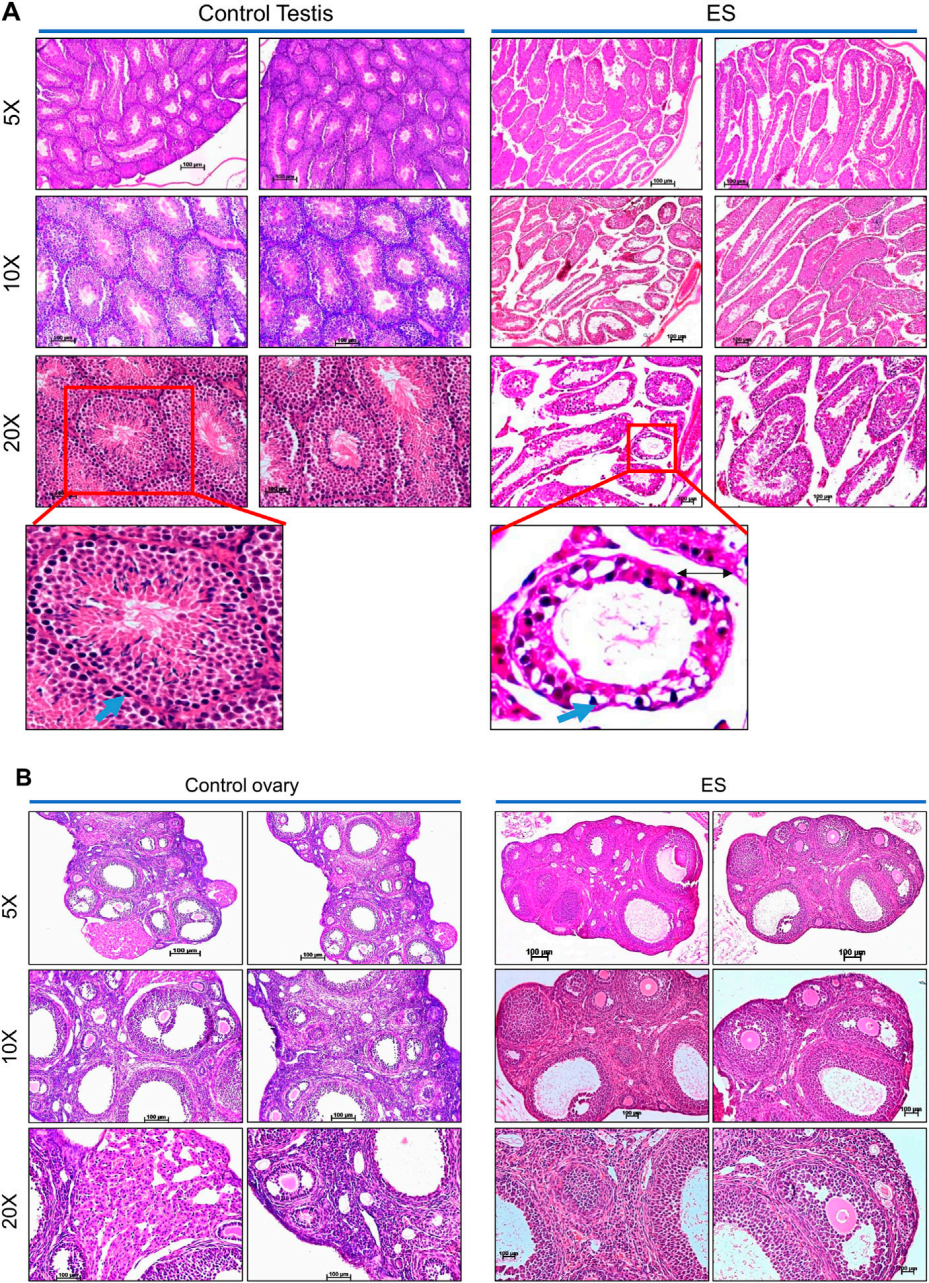
Histopathological examination of reproductive organs from ES-exposed mice. **(A)** Histopathology of testis of mice following ES administration. Control indicates testis tissue from mice with no treatment, and ES represents tissue from ES-treated mice (5 mg/kg, 10 doses; *n* = 2; Magnification: ×5, ×10, and ×20). To evaluate long-lasting effects of exposure to ES, tissue samples were collected after 8 months following exposure. Nuclear components, including heterochromatin and nucleoli were stained by haematoxylin as deep blue or purple while eosin-stained cytoplasmic components like collagen, elastic fibers, muscle fibres and red blood cells. The blue arrows indicate the spermatogonia cells in basal epithelial layer, black arrow in ES indicates the interstitial space between the seminiferous tubules. **(B)** Histopathology of the ovary of mice following ES administration (5 mg/kg, 10 doses) after 8 months of exposure. The experiments were repeated two independent time for each tissue. Control indicates ovary tissue from mice with no ES treatment and ES represents tissue from ES exposed mice (Magnification: ×5, ×10, and ×20; Scale bar: 100 µm).

Histopathological analysis was performed to understand the direct and long-lasting effect of ES on the reproductive organs of exposed mice (Figure 4). After 8 months of ES treatment, analysis of the testes revealed vacuolar degeneration of basal epithelial cells and increased interstitial spaces due to the shrinkage of seminiferous tubules compared to control mice (Figure 4A). Many seminiferous tubules were affected, which had depleted spermatogonia mother cells in ES-exposed animals. A visible difference was also observed in the sperm population in the lumen of seminiferous tubules in treated mice compared to controls (Figure 4A).

Similar to the histopathology of mice testes, decreased follicular activity and an increased number of corpus luteum were observed in the ovary of treated female mice even after 8 months of ES exposure (Figure 4B). An increase in infertility in females correlates with decreased follicular activity in ES-exposed mice ovary. Thus, our results reveal the persistent, long-lasting effect of ES exposure on the male and female reproductive system.

### Endosulfan exposure leads to persistent DNA breaks in the reproductive organs of mice

**FIGURE 5:**
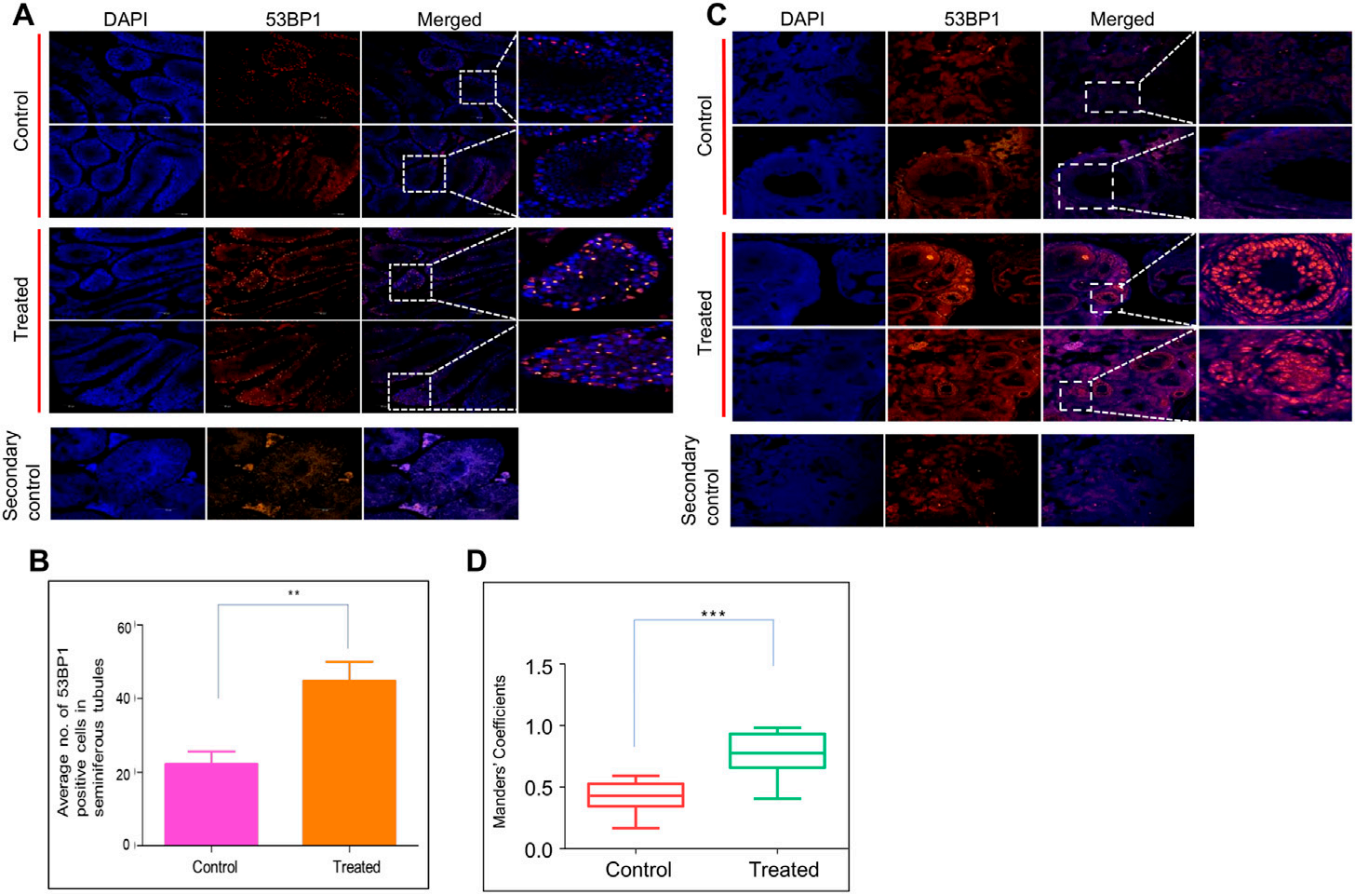
Evaluation of ES induced DNA breaks in mice reproductive organs. To evaluate the long-term effects of ES, mice (*n* = 2) were exposed to ES (5 mg/kg, 10 doses), and tissue samples were collected after 8 months and examined for 53BP1 foci, a hallmark of DSBs. **(A)** Immunofluorescence for 53BP1 expression in testis of ES treated mice. Control indicates sections of testes from untreated control mice, while treated refers to sections from testes of ES-exposed mice. **(B)** Bar graph indicates the average number of 53BP1 positive cells in control and treated mice seminiferous tubules. **(C)** Immunofluorescence of 53BP1 expression in ovary of control and ES treated mice. Control indicates ovary sections from untreated mice, whereas ES is sections from treated mice. The experiments were repeated two independent time. **(D)** Bar graph indicates number of 53BP1 positive cells in control and treated mice nucleus calculated as Mander’s coefficient on ImageJ (ns: not significant, **p < 0.005). In panel **(B)** at least 25 seminiferous tubules and in panel **(D)** at least 20 fields each were analyzed.

Since the histological investigation of reproductive organs implicated the persistent effect of ES, we evaluated the reproductive tissues for long-term persistent DNA damage. DNA damage was investigated by immunofluorescence staining of 53BP1 (p53-binding protein 1) in both testes and ovaries in ES-exposed mice (Figure 5). 53BP1 is a well-known DNA damage response factor, and it is recruited at the site of DNA damage (Gupta et al., 2014). 53BP1 staining on testis sections from ES-exposed mice showed significantly elevated expression in seminiferous tubules of testes when compared to controls, suggesting the generation of DNA double-strand breaks (DSBs) (Figures 5A,B). While the 53BP1 foci were restricted only to the spermatogonial mother cells surrounding the basal epithelial layer of the seminiferous tubules in the control mice testis, a significantly elevated number of 53BP1 foci were seen in spermatocytes and spermatids in the case of treated mice testis. These results implicated the persistent DNA damage in testicular cells when exposed to ES.

Further, a significant increase in 53BP1 foci was observed in the ovary of ES-exposed mice compared to controls (Figures 5C,D). Specifically, 53BP1 foci were seen in primordial follicles and the granulosa cells of the primary follicles in treated mice. Considering that granulosa cells are involved in the maturation of the entire follicle and may play a crucial role in ovarian physiology, damage to these cells affects the proper maturation of the oocytes resulting in infertility (Amsterdam and Rotmensch, 1987).

### Exposure to endosulfan results in overexpression of DNA ligase III in reproductive organs

**FIGURE 6:**
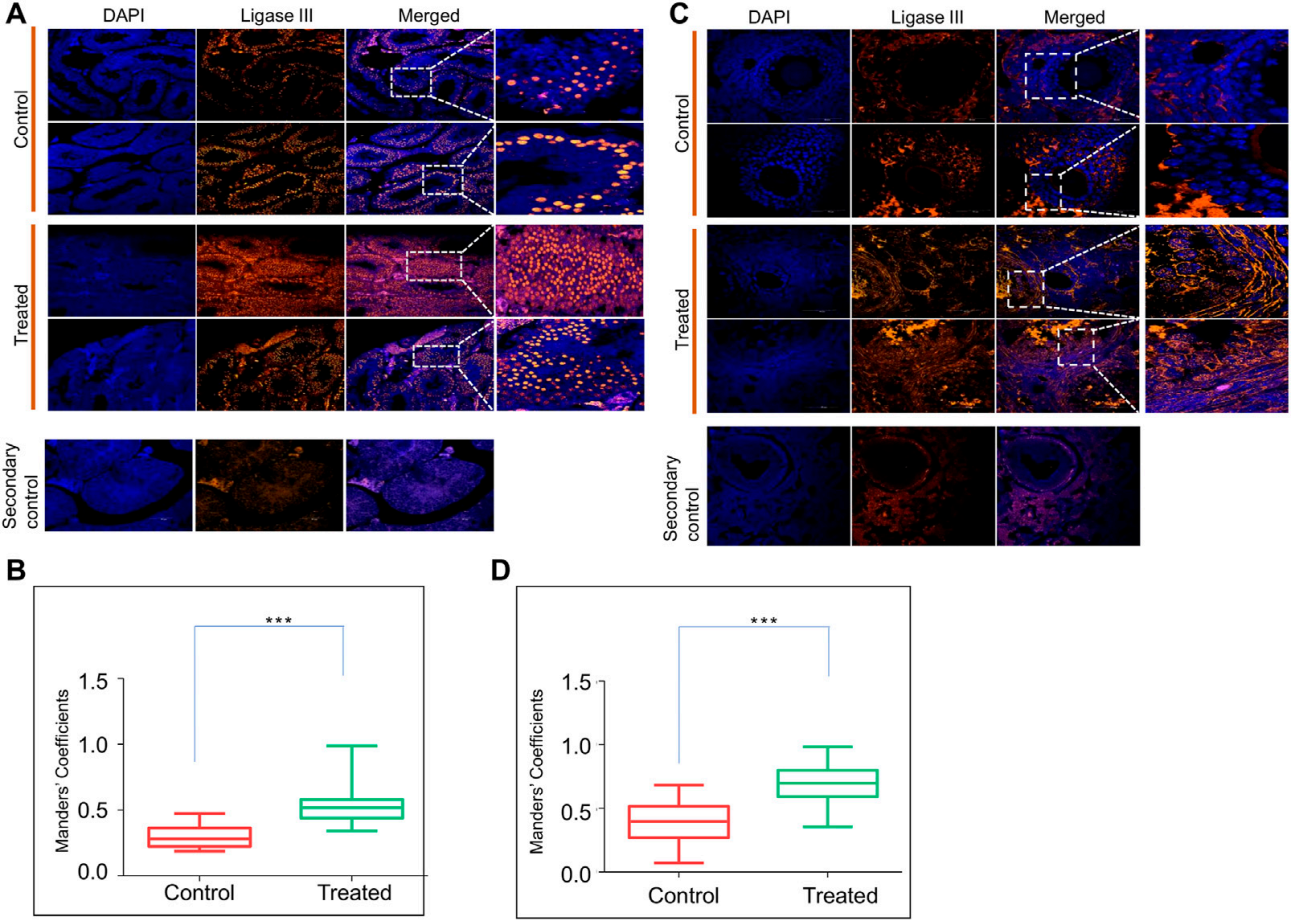
Evaluation of ES induced DNA repair through Ligase III in reproductive organs. **(A)** Immunofluorescence of DNA Ligase III expression in testes of ES-treated mice (5 mg/kg, 10 doses, *n* = 2). Tissue samples were collected after 8 months of exposure to ES. Control indicates sections of testes from untreated mice, whereas, ES is sections from treated mice. The nucleus is counterstained with DAPI. The merged image shows localisation of DNA Ligase III positive cells. **(B)** Bar graph shows localization of DNA Ligase III positive cells in control and treated mice testis calculated as Mander’s coefficient on ImageJ. **(C)** Evaluation of ES induced DNA Ligase III in mice ovary. Immunofluorescence staining of DNA Ligase III expression in ovary of control and ES treated mice. **(D)** Bar graph indicates localization of DNA Ligase III positive cells in control and treated mice ovary calculated as Mander’s coefficient on ImageJ. In panels **(B,D)** at least 20 fields each were analyzed from two independent batches (ns: not significant, *p < 0.05, **p < 0.005, ***p < 0.0001).

Previously, we observed that ES induces error-prone DNA repair through MMEJ in which DNA ligase III is one of the most critical enzymes responsible for the final sealing of breaks (Wang et al., 2005; Sharma et al., 2015; Sebastian and Raghavan, 2016). Therefore, we examined the expression of DNA Ligase III in the reproductive tissues by immunofluorescence staining on the testis and ovary sections after 8 months of ES exposure (Figure 6). In the ES-exposed mice testes, the expression of DNA Ligase III was significantly higher than that of the control (Figures 6A,B). A similar analysis of the ovary section revealed elevated expression of DNA Ligase III in ES-exposed mice (Figures 6C,D). Interestingly, DNA Ligase III expression was observed highest in granulosa cells, as was the case of 53BP1 (Figures 5C,D), which indicated the induction of the alternate error-prone DNA repair pathway. Thus, our results indicate that the persistent ES-induced DNA double-strand breaks in the reproductive organs of exposed mice led to the elevated expression of DNA Ligase III.

### Endosulfan induces damage in the lungs and liver of mice but not in other organs

**FIGURE 7:**
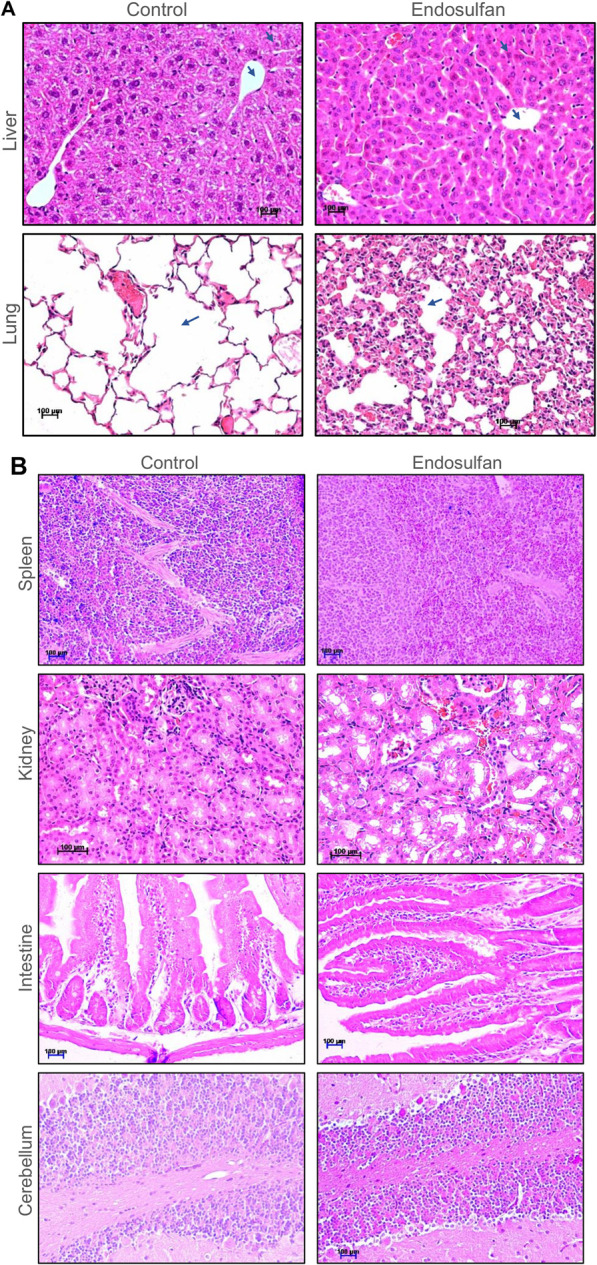
Histopathological examination of vital organs of mice post 8 months of ES treatment. **(A)** Histopathology of liver and lung of mice following ES administration (5 mg/kg, 10 doses). Tissue samples were collected after 8 months of completion of the dose. Control indicates tissue from untreated mice and ES represents tissue from ES treated mice. Hepatocytes and central vein are marked using blue arrows in case of liver. Changes in the architecture of alveoli in lungs is also indicated using arrows. The magnification shown is ×20. **(B)** Histopathology of spleen, kidney, intestine, and cerebellum of mice following ES administration (5 mg/kg, 10 doses). Control indicates tissue sections from untreated mice and ES represents tissue from ES-treated mice. The experiments were repeated two independent time for each tissue. Magnification shown is of ×20. Scale bar: 100 µm.

We were interested in testing the persistence and direct effect of ES on the vital organs of mice after 8 months of its exposure (5 mg/kg, 10 doses). The histopathological analysis of the lungs, liver, kidney, intestine, cerebellum, and spleen was performed on ES-exposed mice after 8 months of the treatment. Results showed changes in the architecture of alveoli, which was more congested in the lungs of treated mice than in control (Figure 7A). Mild vacuolation in hepatocytes was observed in ES-treated mice liver, indicating irritant in the blood system (Figure 7A). We did not find any remarkable difference in the histology of the spleen, kidney, intestine, and cerebellum of the treated mice when compared with the control (Figure 7B). Thus, histopathological analysis revealed that ES-exposure had a persistent effect on the liver, lungs, and reproductive organs.

### Endosulfan induces tumorigenesis in mice

**FIGURE 8:**
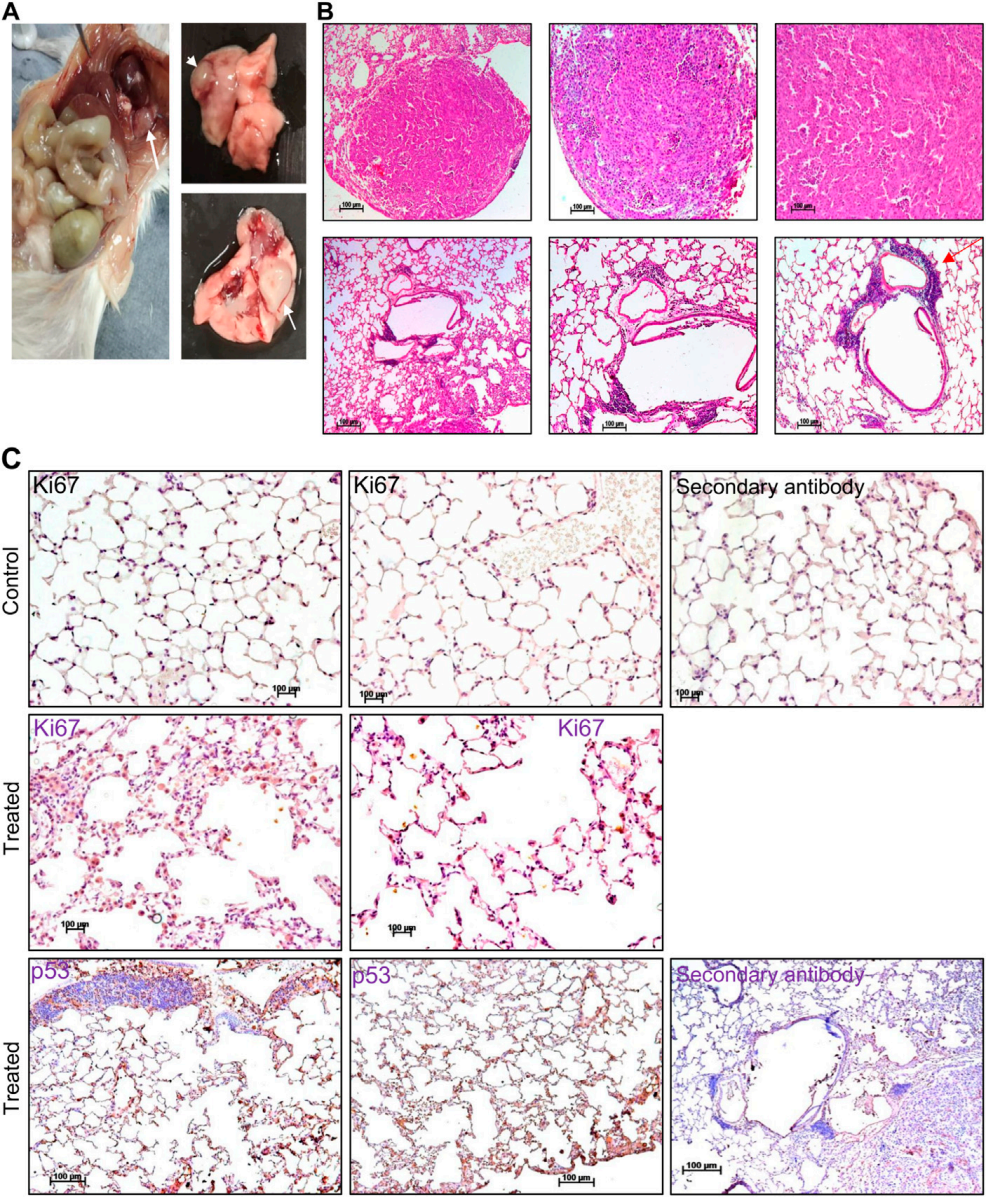
Histopathological examination of abnormal growth in lungs of mice post treatment with ES. **(A)** Abnormal growth was observed in the lungs following 16 months of ES exposure (5 mg/kg, 10 doses) in male mice. The white arrow indicates abnormal growth. **(B)** Histopathological analysis of abnormal growth in lungs from ES exposed male mice. The red arrow indicates the infiltrating cells (×5, ×10, and ×20). **(C)** Immunohistochemical analysis of abnormal growth in lungs of ES exposed mice. Immunohistochemical analysis of control mice lung with Ki67 (top panel) and treated mice lung with Ki67 and p53 (bottom panel) (×20). In panels **(B,C)** experiment was repeated three independent times from the tissues of same animal. Control indicates lung tissue from untreated mice and treated indicates ES-treated tissue from male mice. Scale bar: 100 μm.

**FIGURE 9:**
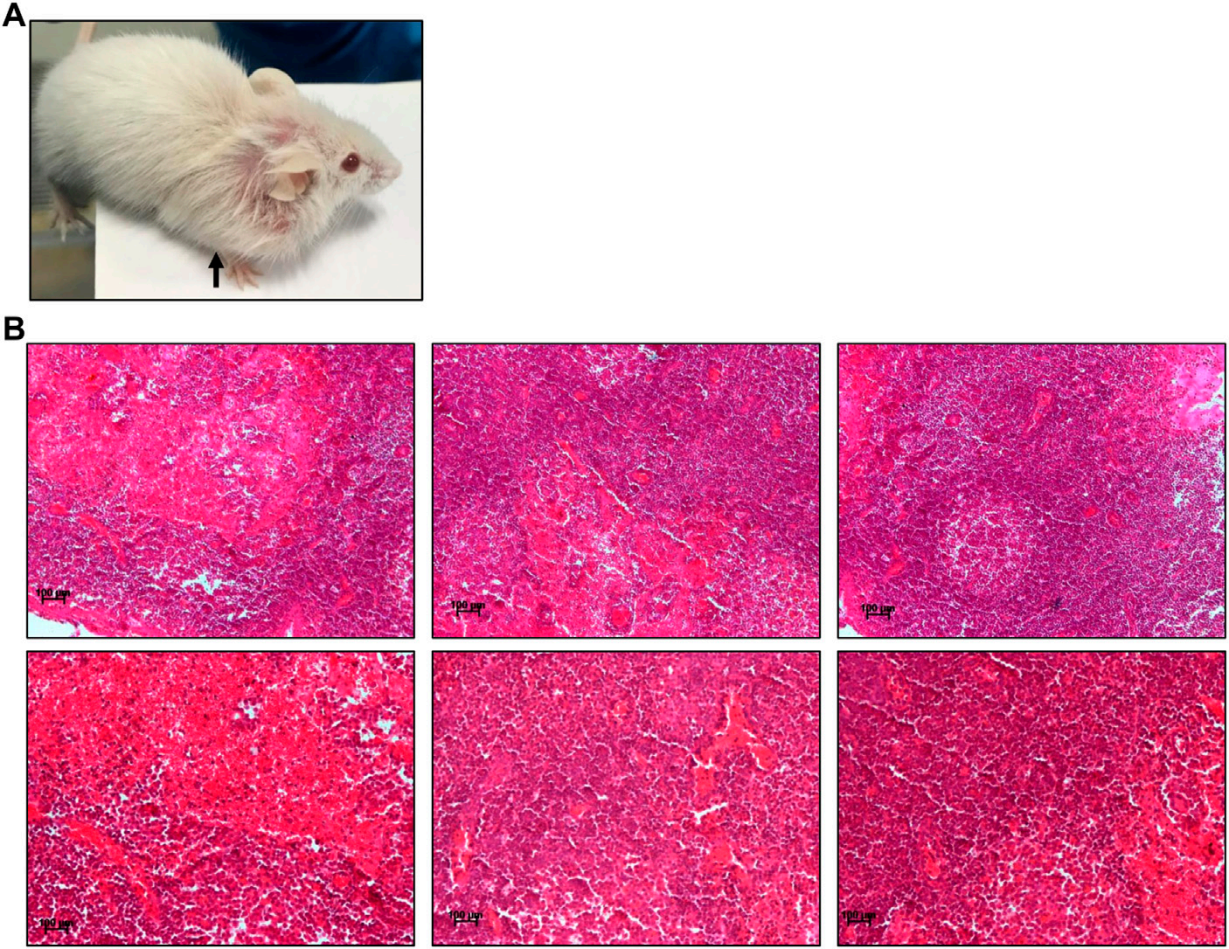
Histopathological examination of abnormal growth in ES-exposed mice post treatment. **(A)** Abnormal growth in neck region observed after 3 months of ES exposure indicated by black arrow in ES-exposed female mice (5 mg/kg, 10 doses). **(B)** Histology of abnormal tissue from the neck region of the ES-exposed female mice (×20). Experiment was repeated three independent times from the tissues of same animal. Scale bar: 100 μm.

Pesticides are considered one of the major factors contributing to increased tumor incidence, although the direct link is still not well established (Anwar, 1997; Dich et al., 1997; Bharathi et al., 2013). In the present study, we observed the development of tumours in ES exposed (5 mg/kg, 10 doses) mice after 3 and 16 months of completion of the dose (Figures 8, 9). Abnormal growth was observed in the lungs of two ES-exposed male mice (Figure 8A, top and bottom panel). Histopathology of the suspected tumour tissues revealed undifferentiated cells (Figure 8B, top panel). Further, infiltrating cells in the bronchiole of the lung were also observed (Figure 8B, bottom panel, marked with a red arrow). To confirm the cancerous nature of the tissues, immunohistochemical analysis was performed with anti Ki67, a cell proliferation marker, and p53, the tumour suppressor gene, in ES-exposed mice. Results revealed the expression of Ki67 and p53 in the lung, confirming that the abnormal growth was indeed cancerous (Figure 8C). We also observed the tumour development in the neck region of one of the ES-exposed female mice 3 months after the dosing (5 mg/kg, 10 doses) (Figure 9A). Further, histopathological analysis of these tissues revealed the presence of undifferentiated cells (Figure 9B). Sheets of neoplastic cells which did not have well-defined borders were also visible. The cells were uniform in size and shape and were mononucleated. Thus, our observations show compelling evidence that ES has the potential to cause long-term harmful effects on organisms, including tumorigenesis.

## Discussion

A preponderance of epidemiology studies shows that pesticides have a health burden on non-target species like humans due to their intrinsic toxicity and limited species selectivity. Widespread pesticide use contaminates the food chain and causes mutagenic activation or inactivation of the ingested chemicals (Pednekar et al., 1987; Bolognesi, 2003; Mostafalou and Abdollahi, 2013; Kapeleka et al., 2021). In the present study, we have used mice as a model system to study the chronic effects of pesticides once exposed when they are 4–8 weeks old. Therefore, the present study can link pesticide exposure and its long-term/persistent impact on humans and provide a molecular basis for its persistent effects.

A distinct increase in infertility and increased reproductive issues have been associated with occupational or environmental exposure to pesticides during the last few decades (Oliva et al., 2001; Joffe, 2003). In the present study, a serum concentration of 50 μg/L (24 h) was used, as this could mimic relevant concentration in such ES-exposed areas. Interestingly, we observed a significant increase in infertility in both male (30%–55%) and female (20%–62%) mice compared to the previous study in which serum concentration was 23 μg/L (24 h) (Sebastian and Raghavan, 2015b). Several studies have also reported that ES affects the reproductive tissues in different animal models (Sinha et al., 1995; Hiremath and Kaliwal, 2002b; Rey et al., 2009; Milesi et al., 2015). The observed increase in infertility in male and female mice was consistent with the epidemiological studies from the affected region (Jayadevan et al., 2005; Kanthila and Shenoy, 2012; Satheesh, 2017; Badiger et al., 2019). It has been shown that ES affects testicular functions due to its effects on reproductive hormones, leading to abnormal spermatozoa and decreased sperm count and sperm motility (Ozmen, 2011; Sebastian and Raghavan, 2015a; Sengupta and Banerjee, 2013). Although there exists a limited amount of human data on the effect of ES, a cohort study by Saiyed et al. (2003) among school children (10–19 years old) showed delayed male sexual maturity and interference with male sex-hormone synthesis due to exposure to ES (Saiyed et al., 2003). ES is also shown to cause ovarian regression in females, alterations in hormone synthesis, follicular maturation, ovulation process, and ovarian cycle, which leads to an increase in infertility (Hiremath and Kaliwal, 2002a; Sharma R. K. et al., 2020).

The effect of ES on the reproductive system is linked to its hormonal-disrupting function, and such pesticides are often called endocrine-disrupting chemicals (EDCs). These EDCs significantly impact the reproductive system; their activity can be due to direct binding with hormone receptors owing to their conformational similarity with receptor-binding portions of natural steroid hormones (Mrema et al., 2013). Numerous studies have shown that ES behaves as an anti-androgen (Singh and Pandey, 1990; Wilson and LeBlanc, 1998; Murono et al., 2001; Viswanath et al., 2010). We have also shown through docking studies and bioinformatic analysis that ES can bind to the ligand-binding site of the androgen receptor with considerable energy compared to its natural ligand dihydrotestosterone in the previous study (Sebastian and Raghavan, 2015a). The reproductive toxicity of ES is not limited to mammals but also extended to various other animals like zebrafish (Han et al., 2011), crocodiles (Tavalieri et al., 2020), *C. elegans* (Du et al., 2015) and Newt (Park et al., 2001) indicating that it could affect most forms of life in the ecosystem.

Besides reproductive organs, the present study revealed that exposure to ES affected the lungs and liver in mice, which was also consistent with our previous results (Sebastian and Raghavan, 2015b). Studies from other animal models have also reported the liver, lungs, brain, and kidney as the major target organ for ES-exposure (Gupta and Chandra, 1977; Seth et al., 1986; Paul and Balasubramaniam, 1997; Caglar et al., 2003; Mor and Ozmen, 2003; Jia and Misra, 2007).

Although acute toxicity studies of pesticides are reported, their long-term effects are not well investigated, particularly in humans. Previously, immediate effects of ES were observed in the testes, lungs, and liver when the histochemical analysis was performed (Sebastian and Raghavan, 2015b). In the present study, when the long-term effect on the vital organs of mice was evaluated, we noted that such effects were persistent. We observed vacuolar degeneration of basal epithelial cells and increased interstitial spaces due to shrinkage of seminiferous tubules in mice testes even after 8 months of ES-exposure. A visible difference in the sperm population was also observed in the lumen of seminiferous tubules in the ES-exposed mice. A decrease in follicular activity in ES-exposed mice ovaries further suggests the pesticide’s long-term effect on female reproductive tissues. A persistent effect was also seen in the lungs and liver of the ES-exposed mice. The architecture of the alveoli of the lungs was changed in ES-exposed mice. In the liver, mild vacuolation in hepatocytes was observed even after 8 months of ES exposure. The only available long-term study conducted in liver tissues of ES-treated mice, which was assessed after 6 months, demonstrated that exposure to ES caused liver tissue damage illustrated by hepatic/somatic index and increased levels of liver Lactate Dehydrogenase (Kurutaş et al., 2006). Further, our results, in conjunction with a previous study, revealed that ES-induced persistent DNA damage in testes and ovaries, altered DNA damage response, and possibly elevated levels of microhomology-mediated end joining (Sebastian and Raghavan, 2016).

A study by the International Agency for Cancer Research (IARC) has reported potential carcinogenicity of chemicals such as phenoxy acid herbicides, 2,4,5-trichlorophenoxyacetic acid (2,4,5-T), lindane, methoxychlor, toxaphene and several organophosphates in laboratory animals but epidemiological data on cancer risk in farmers are conflicting (Bolognesi, 2003). *In vitro* studies have reported the carcinogenic potential of ES (Flodström et al., 1988; Soto et al., 1994; Bulayeva and Watson, 2004; Zhu et al., 2008; Bedor et al., 2010). Besides the general public, farmers and industrial workers are exposed mainly to pesticides. Although there is controversy regarding the carcinogenicity of ES in the exposed region, we report the development of tumours in three different mice exposed to ES several months prior. Two male mice developed a lung tumour, while one female mouse developed a tumour near the neck region. Histopathology and immunohistochemistry studies indeed revealed the existence of cancer cells. Therefore, our study points toward the carcinogenic potential of ES. Further, this was consistent with our previous results, where ES induced an error-prone MMEJ in the reproductive and lung tissues leading to deletions and other genomic rearrangements (Sebastian and Raghavan, 2016). Thus, our study reveals the persistent effect of pesticides and indicates the need for a thorough investigation of the impact of pesticides on human health.

Although the present study is focused on one pesticide, these results can be extrapolated to other pesticides and may be helpful during the policymaking of the use and manufacture of such pesticides. Further, this study also underlines the need for detailed epidemiological and animal studies to understand the extent of pesticide poisoning to human health and the environment.

## Data Availability

The original contributions presented in the study are included in the article/supplementary material, further inquiries can be directed to the corresponding author.
